# Superficial Siderosis After Traumatic Brain Injury: A Case Report

**DOI:** 10.7759/cureus.55314

**Published:** 2024-03-01

**Authors:** Brian G Nudelman, Marianne Cortes, Aditya Sapasetty, Raphael Khella, Danielle Katz

**Affiliations:** 1 Internal Medicine, Memorial Healthcare, Pembroke Pines, USA; 2 Osteopathic Medicine, Nova Southeastern University Dr. Kiran C. Patel College of Osteopathic Medicine, Davie, USA; 3 Internal Medicine Residency, Broward Health Medical Center, Fort Lauderdale, USA; 4 Internal Medicine Residency, Florida Atlantic University Charles E. Schmidt College of Medicine, Boca Raton, USA; 5 Dr. Kiran C. Patel College of Osteopathic Medicine, Nova Southeastern University, Fort Lauderdale, USA; 6 General Surgery Residency, Spectrum Health, Grand Rapids, USA

**Keywords:** traumatic brain injury, subarachnoid, neurosurgery, tinnitus, ataxia, hemosiderin, mri, neuroradiology, siderosis, superficial siderosis

## Abstract

Superficial siderosis (SS) is a rare condition in which chronic accumulation of the blood in the subarachnoid space over time leads to the buildup of hemosiderin deposits, which in turn cause neurological dysfunction in those affected. While reversibility of the damage done by this condition is nearly impossible, early detection can allow for immediate surgical intervention and thus prevent further progression of ataxia, hearing loss, and other neurological deficits caused by SS. We present a case of a 53-year-old male who was successfully diagnosed with SS secondary to a chronic post-traumatic pseudomeningocele and underwent surgical repair with the resolution of his symptoms. We aim to encourage more extensive workups for common neurological dysfunctions such as tinnitus or vertigo in patients who have a history of traumatic brain injury or any significant motor vehicle accidents.

## Introduction

Superficial siderosis (SS) is a rare condition resulting from the chronic leaking of blood into the subarachnoid space leading to a buildup of hemosiderin in the brain. The pathogenesis of superficial siderosis is believed to be that the deposition of hemosiderin causes the brain to secrete ferritin, creating an inflammatory process leading to neuronal damage [1,2]. In addition, hemoglobin breakdown results in a heme by-product, which activates heme oxygenase-1 to produce iron, leading to neurotoxic buildup. Furthermore, iron buildup results in neuronal damage due to free radical production and lipid peroxidation. A few common etiologies of superficial siderosis include dural defects, central nervous system (CNS) tumors, cerebrovascular trauma, radiation therapy, and disc disease [1-3]. A study in 2017 by Pichler et al. showed an increased prevalence of SS in those 70 years of age or older that was likely associated with the natural increase in deposition of amyloid in the brain, causing cerebral amyloid angiopathy. Additionally, they found a correlation between those with the apolipoprotein E2 allele and the development of SS [4]. Cortical superficial siderosis (cSS) is generally associated with cerebral amyloid angiopathy. It differs in presentation from classical superficial siderosis in the sense that it is usually supratentorial rather than infratentorial and has transient episodes of neurological dysfunction [5]. 

Common symptoms of superficial siderosis include sensorineural hearing loss, gait ataxia, and myelopathy. Diagnosis of superficial siderosis is typically made with contrast-enhanced magnetic resonance imaging (MRI) of the brain, and the finding of hemosiderin deposition strongly indicates SS. For further investigation, magnetic resonance (MR) myelography can be used to identify dural tears [1]. There are several treatment options available that can slow the progression of the disease or offer symptomatic relief. If a dural defect is found, surgical repair can be done to prevent further leakage. Symptomatic treatment of superficial siderosis tends to focus on the improvement of hearing loss and ataxia [2]. The prognosis of this disease depends on the extent of the irreversible brain damage the patient has suffered as well as the accessibility to repair their dural defect [1-3]. The following report aims to bring more awareness to SS as a possible differential diagnosis in those with a prior history of motor vehicle accidents (MVAs) who present with nonspecific neurological symptoms.

## Case presentation

A 53-year-old African American male with a past medical history of hypertension and right upper extremity (RUE) paralysis after a MVA presented to the emergency department with a three-day history of worsening vertigo. Of note, the patient had been to multiple emergency rooms over the past few years with the same complaint, and multiple CT brains returned negative for any abnormalities. This time, the vertigo worsened concurrently with a recent viral upper respiratory illness. He had associated headaches, fatigue, tinnitus in the right ear, and multiple episodes of non-bilious, non-bloody vomiting. The patient endorsed that his symptoms were worsening every day, so he decided to come to the emergency department. He denied fever, blurred vision, or confusion. He claimed he has had many episodes of vertigo associated with vomiting since childhood. He had a history of a traumatic brain injury 30 years prior, after a MVA in Jamaica, which resulted in loss of consciousness and a right upper brachial plexus injury causing complete loss of movement and sensation of the right upper extremity. He endorsed a history of hypertension but denied any medication use. He denied any surgical history or use of tobacco products, illicit drugs, or alcohol. 

On examination in the emergency room, the patient was afebrile, normotensive, and saturating well on room air. He was given meclizine 25 mg. He appeared to be in mild distress but alert and oriented to person, place, and time, and the Glasgow Coma Scale (GCS) was 15. Neurological examination showed no focal deficits bilaterally in the lower extremities nor the left upper extremity. He was unable to move his right upper extremity (RUE), and the Medical Research Council (MRC) Scale was 0/5 for strength in the RUE and 5/5 for the rest of his extremities. He also noted a total lack of sensation when touched. His pupils were equal and reactive to light; the Mini-Mental State Examination (MMSE) showed normal cognitive function; the otoscopic exam showed a normal tympanic membrane and no abnormalities. Upon evaluation, there was no dysmetria; however, the patient had a broad-based, unsteady gait. Orthostatic vitals were negative. A CT of the brain without contrast showed no acute abnormalities. Subsequently, the patient was admitted to the internal medicine team for further evaluation. ENT was consulted due to tinnitus and hearing impairment in the right ear. They placed the patient on 10 mg of methylprednisolone and valacyclovir 1 g three times daily. Unfortunately, there was no improvement in the patient’s symptoms after 48 hours.

**Figure 1:**
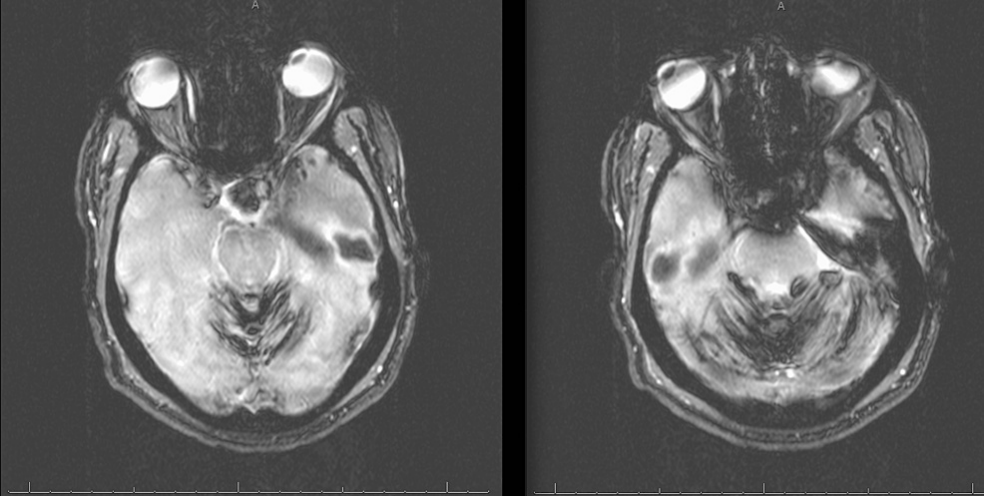
MRI with intravenous contrast of the brain: diffuse hemosiderin deposits in the cerebellar hemispheres bilaterally suspicious for superficial siderosis and hypointensities on plial surfaces.

**Figure 2:**
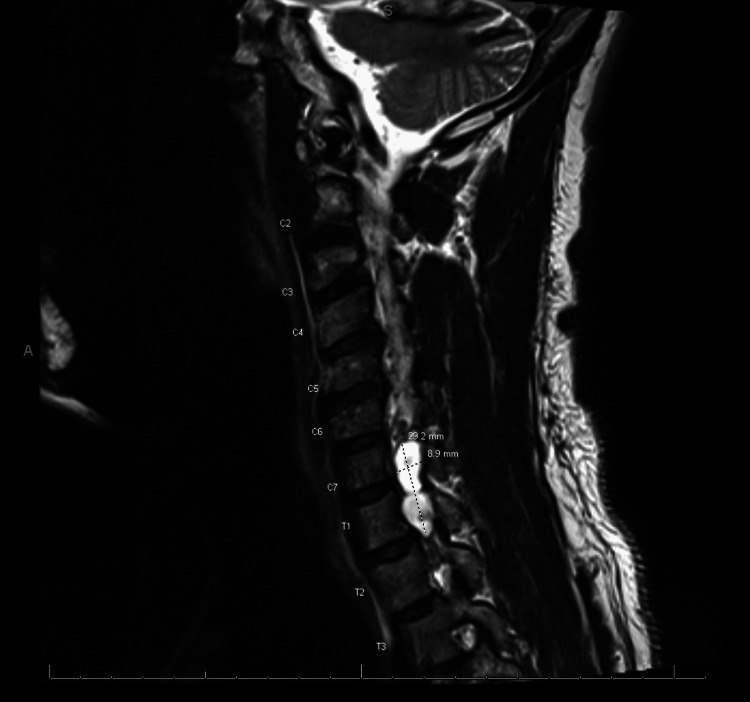
MRI with intravenous contrast of the cervical spine: right-sided pseudomeningocele in the cervical canal at C7 and T1. Non-visualization of the exiting C8 and T1 nerve root, which is consistent with a nerve root avulsion.

An MRI with contrast of the brain was performed, which showed diffuse hemosiderin deposits in the cerebellar hemispheres bilaterally and was suspicious for superficial siderosis (Figure 1). Following this, neurosurgery was consulted, and an MRI of the cervical spine was performed, showing a right-sided pseudomeningocele in the cervical canal at C7 and T1 (Figure 2). There was non-visualization of the exiting C8 and T1 nerve roots, which is consistent with a nerve root avulsion. 

A lumbar puncture was performed and showed colorless cerebrospinal fluid (CSF) with 48 red blood cells. The patient underwent a cervical hemilaminectomy with the repair of a pseudomeningocele through a multilayer fascial closure. He tolerated the procedure well and had minimal blood loss. The patient still had his baseline deficits in the RUE as well as no change in his tinnitus; however, he recovered well. The patient’s balance difficulties were resolved, and the patient no longer had a broad-based gait. He was discharged home with no medications following a physical therapy assessment but was educated about the importance of maintaining close follow-up with neurosurgery on an outpatient basis.

## Discussion

Effective management of superficial siderosis is dependent on the etiology of the disease. In our case, we were able to successfully treat a case of superficial siderosis through the repair of a pseudomeningocele that formed due to a MVA many years prior. In dural defect cases, the onset of symptoms happens slowly over a long period of time; however, surgical repair can slow the progression of SS by patching the leak [2]. Clinically, the effects will vary on a case-by-case basis, but the goal is to offer symptomatic relief for patients. It is important that the patient maintains long-term follow-up to ensure that their condition is stable, as this condition progresses. Similarly to the patient presented in this case, it is difficult to recover or reverse the neurologic symptoms such as ataxia or difficulty hearing; they may benefit from physical therapy to allow for improvement. As for their hearing, there have been cases where patients have reported improvement after receiving a cochlear implant. Finally, there have been attempts to see if using chelation in the form of agents like deferiprone can provide a clinical benefit. Nevertheless, there has been wide variation in response, with some patients reporting improvement while others have reported further worsening of their symptoms [1-3]. 

Not all cases of SS have an insidious progression, and some can happen rather quickly. A recent case report by Friedauer et al. mentioned a phenomenon called “superficial siderosis syndrome." In this case, the patient developed rapid-onset superficial siderosis due to a hemorrhagic brain mass. The effective treatment in this situation actually involved surgical removal of the offending mass, and similarly to the more insidious versions of superficial siderosis, the patient in that case did not recover the neurologic function he had already lost. However, the surgery did stop further progression of neurological damage, and the patient was eventually discharged home [6]. 

In the seven years prior to this hospitalization, our patient made over five visits to the ED with complaints of intermittent dizziness that varied in intensity. The extent of these workups included CT images of the brain on two different occurrences that both showed no abnormalities. Contrast-enhanced MRIs were not thought to be warranted until his most recent hospitalization, which included complaints of hearing impairment. The image results showed hemosiderin deposits. These findings, in addition to the patient’s history of MVA that resulted in a permanent brachial plexus injury, called for subsequent follow-up imaging of the cervical spinal cord. An MRI of the cervical spine was significant for a pseudomeningocele in the cervical canal. Neurosurgical intervention repaired the abnormality, and the patient’s gradual neurological decline has since come to a halt. Given this course, it is highly likely the pseudomeningocele was the ultimate cause of this chronic hemosiderin deposit. This would place our patient's presentation in the “classical” rather than cortical superficial siderosis (cSS) category. This means that his SS likely stemmed from a slow leak of blood into the spinal cord or infratentorial space rather than from an abnormal supratentorial vessel structure secondary to cerebral amyloid angiopathy [5,7]. Also, cSS often presents with transient neurological episodes known as amyloid spells rather than constant progressive neurological dysfunction, as seen in our patient [8]. The presentation of this case is important because neurological deficits secondary to siderosis are usually irreversible, making early diagnosis with an MRI imperative to improving patient outcomes.

While we understand the reservations about ordering MRIs due to the increased cost to patients and length of hospital stay, we urge that it be highly considered for those with a history of traumatic brain injuries or MVA. This may be difficult at institutions with limited resources; however, in similar cases, we urge consideration of the immediate transfer of patients to an MRI-capable center if possible. While this report only illustrates a single case of classical SS and does not have a large statistical sample, we believe that the patient presentation is important and can help raise suspicion for underlying dural defects in patients with similar clinical pictures.

## Conclusions

Superficial siderosis is a rare but neurologically devastating condition. As demonstrated in our patient, the standard workup for patients presenting with complaints of vertigo is not adequate to diagnose SS effectively. This case demonstrated how multiple CT scans over various hospital visits missed an underlying dural defect in the cervical spinal cord. While we do not recommend MRI brain and cervical spine and MR myelography for every patient who comes to the hospital with complaints of vertigo or tinnitus, we do want to draw attention to the indications for further workup in those with a history of traumatic brain injury. Our patient's motor vehicle accident with subsequent chronic vertigo was a significant risk factor that should have prompted an earlier extensive workup with an MRI. Early diagnosis and treatment of SS through neurosurgical intervention can prevent the worsening of irreversible symptoms such as hearing loss and ataxia.
